# Three‐dimensional mapping of the joint space for the diagnosis of knee osteoarthritis based on high resolution computed tomography: Comparison with radiographic, outerbridge, and meniscal classifications

**DOI:** 10.1002/jor.24015

**Published:** 2018-04-27

**Authors:** Houda Mezlini‐Gharsallah, Rabaa Youssef, Stéphanie Uk, Jean D. Laredo, Christine Chappard

**Affiliations:** ^1^ B2OA UMR 7052 CNRS Paris Diderot University 10 Avenue de Verdun 75010 Paris,Sorbonne Paris Cité Paris France; ^2^ CEA Linklab Site El Ghazala Technopark 2088 Ariana Tunis Tunisia; ^3^ COSIM, Carthage University Carthage Tunisia; ^4^ Radiology Department Hospital Lariboisière 2 Rue Ambroise Paré 75475 Paris Cédex 10, Sorbonne Paris Cité France

**Keywords:** knee, cartilage, osteoarthritis, 3D computed tomography, biomarkers

## Abstract

One of the most important characteristic of knee osteoarthritis (OA) is the joint space (JS) width narrowing. Measurements are usually performed on two dimensional (2D) X‐rays. We propose and validate a new method to assess the 3D joint space at the medial knee compartment using high resolution peripheral computed tomography images. A semi‐automated method was developed to obtain a distance 3D map between femur an tibia with the following parameters: volume, minimum, maximum, mean, standard deviation, median, asymmetry, and entropy. We analyzed 71 knee specimens (mean age: 85 years), radiographs were performed for the Kellgren Lawrence (KL) score grading. In a subgroup of 41 specimens, the histopathological Outerbridge and meniscal classifications were performed and then cores were harvested from the tibial plateau in three different positions (posterior, central, and peripheral) and imaged at 10 µm of resolution to measure the cartilage thickness. Minimum, maximum, mean, and median were statistically lower and entropy higher between knee specimens classified as KL = 0 and KL = 3–4. Gr1 and 2 were statistically different from Gr3‐4 for minimum, asymmetry, entropy using the Outerbridge classification and Gr1 was statistically different from Gr3–4 using the meniscal classification. Asymmetry, minimum, mean, median and entropy were significantly correlated with cartilage thickness. Parameters extracted from a 3D map of the medial joint space indicate local variations of JS and are related to local measurements of tibial cartilage thickness, and could be consequently useful to identify early OA. © 2018 The Authors. *Journal of Orthopaedic Research*® Published by Wiley Periodicals, Inc. on behalf of Orthopaedic Research Society. J Orthop Res 36:2380–2391, 2018.

**Abbreviations**
2D: two‐dimensional
3D: three‐dimensional
CT: computed tomography
HR‐pQCT: peripheral computed tomography
JSW: joint space width
KL: Kellgren Lawrence grades
MRI: magnetic resonance
OA: osteoarthritis
VOI: volume of interest

Knee OA presents the greatest morbidity and commonly affects the medial compartment.^1^ The radiographic abnormalities in OA have been described extensively in an atlas.^2^ The main characteristics are the narrowing of the joint space width (JSW), the sclerosis of the subchondral bone, and the presence of osteophytes.^2^ In order to follow the natural history of OA or the effect of new drugs, a common grading system is used to assess the severity of knee OA using five grades from normal to severe.^3^ It is also possible to make an indirect measurement of the cartilage thinning on posterior‐anterior standing X‐rays by measuring the JSW between the articular cortices of the femur and the tibial plateau. This method is still recommended to assess the structural disease progression in clinical trials by regulatory authorities.^4^ Indeed, a 0.1‐mm reduction over 3 years of JSW was associated with a 14% increased risk for knee replacement.^5^ This measurement can be done manually with a lens and a rule.^6^, ^7^ by semi‐automated methods, or by fully automated methods on digital radiographs.^8^, ^9^, ^10^, ^11^

The knee joint is a complex structure, and the development of OA imaging biomarkers could lead to a better understanding of the natural history of OA and its treatment mechanisms.^12^ On two‐dimensional (2D) radiographs, the reliability and precision of the JSW measurement are dependent on the acquisition conditions, such as the position of the knee, the knee bending angle, and the tibial plateau alignment with the X‐ray beam.^13^ Consequently, it is important to extract the 3D information of JS. Digital X‐ray tomosynthesis has been tested, this technique has the potential to limit the superimposition of the soft tissue but presents a high anisotropic resolution leading to different behaviors of the JS measurements between the posterior–anterior and lateral views.^14^ Imaging by 2D multiple planes or 3D structural information of the knee joint can be provided by magnetic resonance imaging (MRI) and computed tomography (CT). The technique of MRI is a widely used modality to visualize cartilage, joint effusion, ligaments, tendons, meniscus, osteophytes, and bone marrow lesions.^15^ In clinical research, different approaches are developed using MRI images for diagnosis and follow‐up of OA: semi‐quantitative scorings, quantitative assessment of the cartilage volume, or evaluation of compositional cartilage with the main advantage to be radiation free.^13^ Semi‐quantitative scorings are usually performed on 2D sequences and 3D sequences having near isotropic resolution, in order to measure the cartilage volume.^13^ Segmentation of cartilage can be manual,^16^ or fully automatic.^17^ However, the 3D sequences are very time consuming and can appear blurred; therefore these sequences are not able to reveal details about other important joint structures.^13^ Moreover, the use of 3 Tesla machines is often necessary to obtain good signal to noise ratio.^13^

Imaging by CT is not used in routine clinical investigations of knee disease because a significant radiation dose is delivered; however, it is known to provide excellent visualization of bone and calcified tissue, and its use has been proposed to evaluate calcium deposition,^18^ quantitative analysis of regional bone mineral density,^19^ and semi‐quantitative scoring of cartilage lesions with arthrography.^20^ High resolution peripheral QCT (HR‐pQCT) images are clinically used to study separately trabecular bone density and micro‐architecture and cortical bone density, thickness, and porosity.^21^ Recently, new developments have been performed to study bone micro‐architecture of the human knee in vivo.^22^

The aim of this study is to determine if the quantification of the local variation of JSW in three dimensions using HR‐pQCT images is able to reflect cartilage and meniscal degradation.

## METHODS

### Knee Specimen Description

Seventy‐one knee specimens were collected at the Institute of Anatomy Paris Descartes (from 44 females and 27 males aged from 58 to 101 years; mean age: 84.7 ± 9.9). The collection of these human tissue specimens was conducted according to pertinent protocols established by the Human Ethics Committee at Inserm. Due to this regulation, no data were available regarding the cause of death, previous illnesses, or medical treatments of these individuals. After soft tissue removal, knee specimens were stored at −20°C.

### X‐Ray Imaging

All specimens were radiographed in the posterior‐anterior position with an Axiom Luminos Siemens^®^ apparatus to define the Kellgren Lawrence (KL) scoring.^3^ Knees with KL = 0 were considered as normal, KL = 1 as early OA, KL = 2 as moderate OA, and KL > 2 as late OA.

### High Resolution CT Imaging

All the knee specimens were scanned using high‐resolution peripheral quantitative computed tomography (HR‐pQCT) XtremeCT Scanco^®^ Medical Brüttisellen, Switzerland. Usually, this device is used to measure bone mineral density (BMD) and the trabecular bone micro‐architecture at the tibia and the radius for separately assessing the trabecular and cortical bone, with the aim of detecting bone fragility.^21^ The scan (60 KvP, 900 μA) provided high‐resolution images with a nominal isotropic voxel size of 82 μm. Height contiguous scans were necessary for each knee which required 20 min to generate a stack of 976 grayscale reconstructed images with 1536 × 1536 pixel size. Each rotation delivered less than 5 mSv.^23^

### Histopathological Grading and Cartilage Analysis

A subgroup of knee specimens (*n* = 41, from 16 males and 25 females with a mean age of 81.9 ± 10.1 years) were dissected, and the tibial plateaus were excised parallel to the joint surface. To improve cartilage visualization, especially cartilage fibrillation, joint surfaces were stained with waterproof black India ink (Sanford Rotring, Hamburg, Germany).^24^ The modified Outerbridge classification was used to assess the grade of cartilage degradation: grade 0, normal cartilage; grade 1, cartilage softening and swelling; grade 2, mild surface fibrillation and/or loss of cartilage less than 50% of the cartilage thickness; grade 3, severe surface fibrillation and/or loss of more than 50% of the cartilage thickness; and grade 4, complete loss of cartilage with subchondral bone exposure.^25^ The meniscal classification has been previously described.^26^ Briefly, it is as follows: Gr1, normal intact menisci attached with sharp inner borders; Gr2, fraying at inner borders, surface fibrillation, and no tears; Gr 3, partial substance tears, fraying, and fibrillations; Gr4, complete substance tear and loss of tissue.

**Figure 1:**
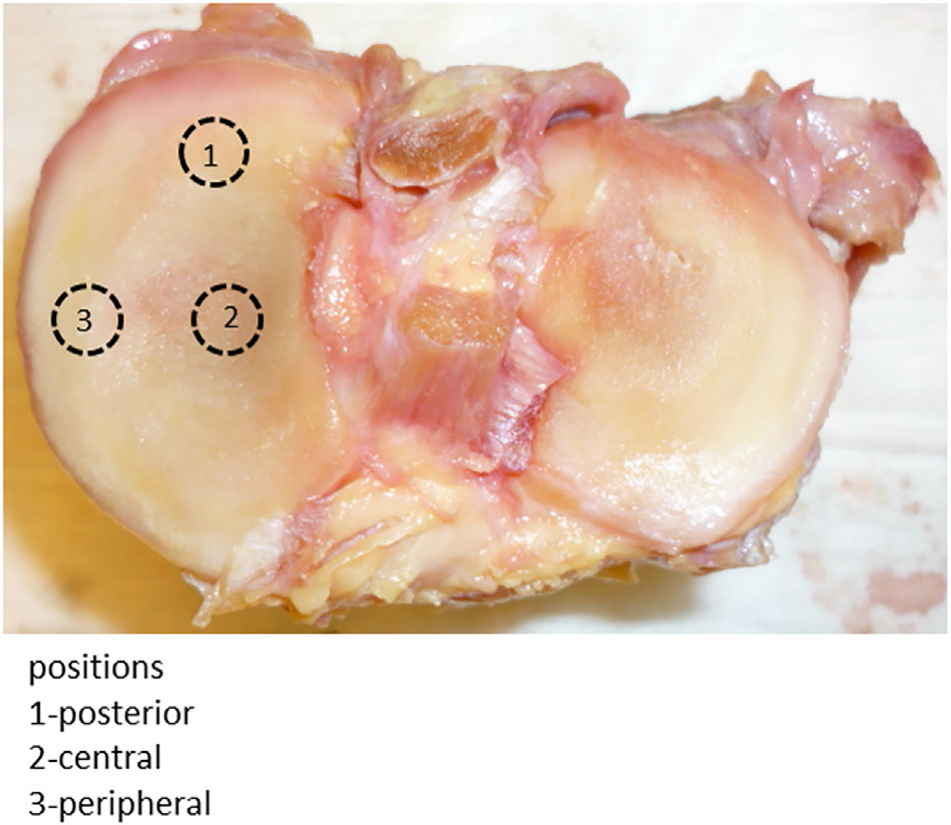
Tibial plateau without meniscus and osteochondral plug sampling sites.

Three bone cores (3 cm height and 7 mm diameter) were harvested with a circular diamond saw (BROT^®^, Argenteuil, France) in the medial tibial plateau in three different positions: (i) peripheral cores located midway between the anterior and posterior edges of the tibial plateau in an external position totally covered by meniscus, (ii) medial anterior cores located in an central position compared with the lateral core never covered by meniscus, and (iii) medial posterior cores located in an internal position compared with the lateral core and in a more posterior position (POST) and partially covered. The different positions are illustrated in Figure 1. The cores were imaged with a micro‐computed tomography system (Skyscan 1172^®^) at 37 kV and 100 μA with a voxel size of 10.2 μm. The details have been described elsewhere.^27^ Cartilage thickness was measured by the sphere method after manual contouring of the cartilage.^28^

### Joint Space Segmentation

To analyze the JSW, a semi‐automated method combining three image‐processing steps has been developed. The complete process has been described in detail elsewhere and briefly described in the present paper.^29^

**Figure 2:**
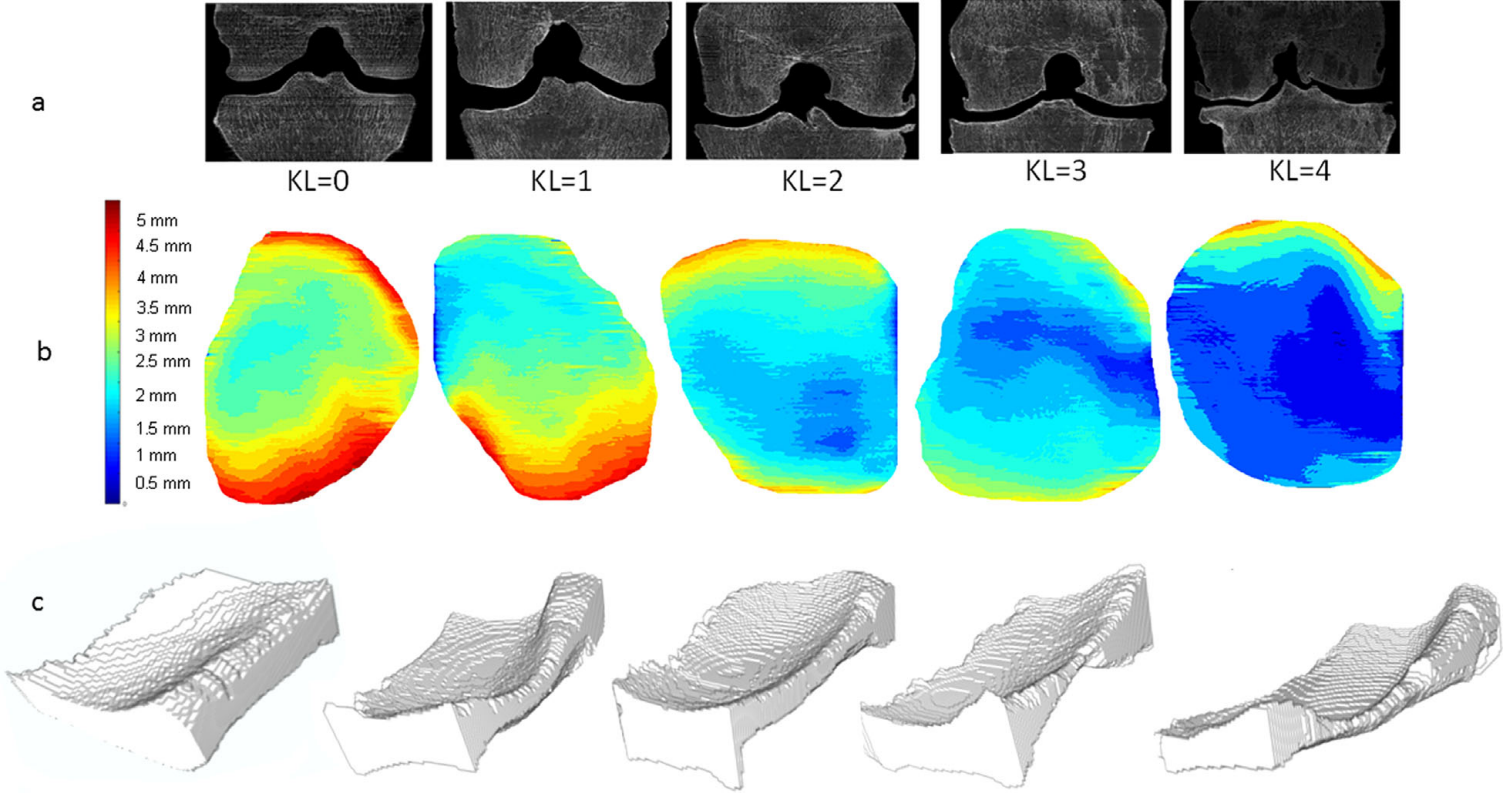
(a) Middle coronal slices of the VOI for different knee specimens with various KL classifications are represented with their corresponding maps. (b) 3D map of medial compartment with color scale from 0 to 5 mm. (c) The 3D joint space mask.

In order to standardize the knee position, the specimen was reoriented by the user so that the tangent to the bi‐condylar femoral posterior line was positioned to be parallel to the X‐axis. For smoothing the image and enhancing edges, an average filter was applied to the original CT DICOM grayscale images with a disk of eight pixels; thus, it was a possible to find a stable value of gray levels for separating bone from soft tissue for the whole stack of images. Following this binarization step, morphological closing, and opening operations (25 pixels, equivalent to 2 mm) were applied to reconnect all bone regions and to fill holes. To eliminate any remaining residual tissue or noise on the coronal slices a 3D mask was created using a 3D hysteresis threshold, and 3D closing operators and 2D connectivity criterion were used to select the largest mutually connected object (i.e., bone). The obtained binary 3D image was then masked with the 3D coronal volume to keep only the bone voxels. The selection of the VOI was carried out manually by the user who defined the number of coronal slices and the internal limit of the medial compartment positioned at the origin of the tibial spine. The external limit was drawn automatically by a tangent line. Next, the joint space edges were automatically determined using the active contour. On the middle coronal slice of the VOI, the user drew 25 control points in the region corresponding to the joint space to initiate the application of the snake model to the entire VOI. The JSW was calculated as the Euclidian distance between the femur and tibial margins at each point. The resulting map represents the distribution of local width measurements through the VOI. Different examples of maps from KL = 0 to KL = 4 with their respective coronal CT images corresponding to the middle of the joint space are represented (Fig. 2).

The JS volume (JS_Vol, mm^3^) was calculated by counting all the voxels inside the segmented VOI. From the 3D map, the following morphometric parameters were calculated: mean (JS_mean, mm), median (JS_median, mm), minimum (JS_min, mm), maximum (JS_max, mm), and standard deviation (JS_SD, mm). The ratio of the maximum JS and the minimum JS defines the JS asymmetry (JS_asym),^23^ and joint space entropy (JS_ent) quantifies the homogeneity of the JS distribution.

JS_ent is calculated according to the following formula:

(1) $$JS\_ent=-\sum_{i=1}^{n}p_{i}\log_{2}\left(p_{i}\right)$$

with *p_i_* the probability to have the value *i*. If the entropy is high then the distribution of the JS presents large local variations.^30^

### Statistical Analysis

The reproducibility was tested because of different manual operations (for example, selection of the threshold value, definition of the number of coronal slices, and initiation of the snake model). For this test, two users, one skilled and one unskilled, performed the different steps on 10 knees with various KL scores, and the reproducibility was assessed by the root mean square deviation (RMSD) for all JS parameters measured.^31^

In each KL measurement (*n* = 71), Outerbridge and meniscal classification (*n* = 41), the parameters extracted from the JS 3D map of knee specimens from males and females were compared by a *t*‐test.

One‐way analysis of variance (ANOVA) followed by a post‐hoc analysis (Bonferroni test) for multiple comparisons were used to investigate group differences in morphological JSW measurements according to the KL, Outerbridge, and meniscal classifications for both genders. In the case of non‐normal distributions a Kruskall–Wallis test was used instead.

A Kruskall–Wallis test was used to compare the JSW distributions (JSW_1–2 mm_, JSW_2–3 mm_, JSW_3–4 mm_, JSW_>4 mm_) found in the different KL grades in the subgroup of specimens from males and females.

Pearson correlation coefficients were used to compare in situ measurements of the cartilage thickness and parameters extracted from the 3D maps.

## RESULTS

The RMSD was 0.03 and 0.17 mm for JS_min and JS_max, respectively, and it was 0.01 and 0.02 mm for JS_mean and JS_SD, respectively. The RMSD was 0.21 and 0.006 for JS_asym and JS_ent, respectively.

Based on the KL classification there was no difference between males and females, and based on the Outerbridge classification there was no difference between males and females, except for Grade 2: JS_mean (*p* = 0.03), JS_asym (*p* = 0.02).

**Figure 3:**
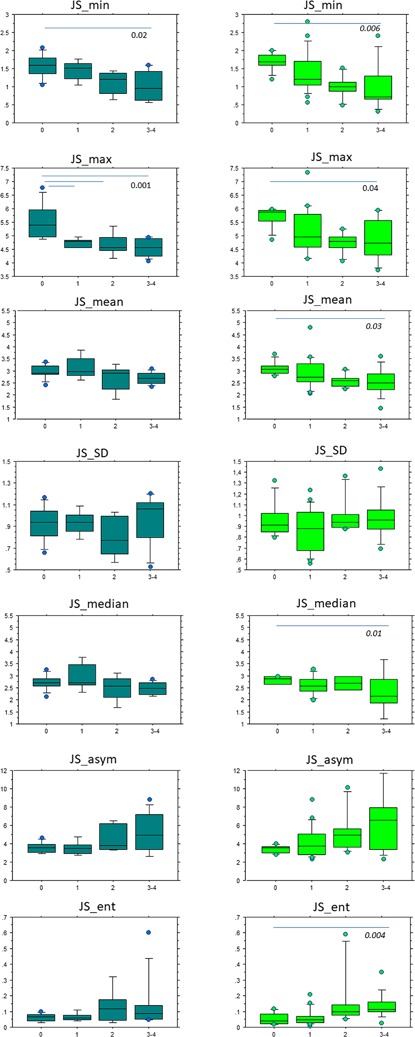
Box plots of JS_min, JS_max, JS_mean, JS_SD, JS_median, JS_asym, JS_ent values according to the KL grades of males (right): KL = 0 (*n* = 8), KL = 1 (*n* = 5), KL = 2 (*n* = 5), KL = 3–4 (*n* = 9) and females (left): KL = 0 (*n* = 8), KL = 1 (*n* = 19), KL = 2 (*n* = 6), KL = 3–4 (*n* = 11).

**Figure 4:**
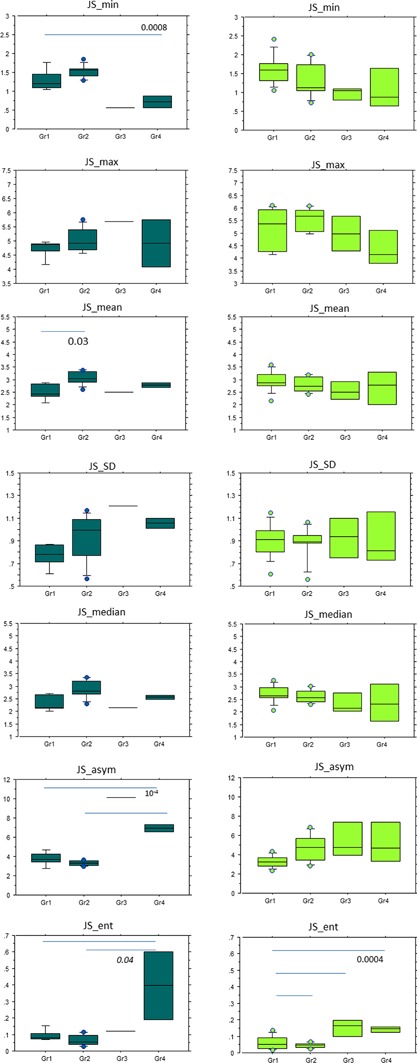
Box plots of JS_min, JS_max, JS_mean, JS_SD, JS_median, JS_asym, JS_ent values according to the Outerbridge classification of males: Gr1 (*n* = 5), Gr2 (*n* = 9), Gr3 (*n* = 1), Gr4 (*n* = 2) and females (left): Gr1 (*n* = 11), Gr2 (*n* = 7), Gr3 (*n* = 3), Gr4 (*n* = 4).

**Figure 5:**
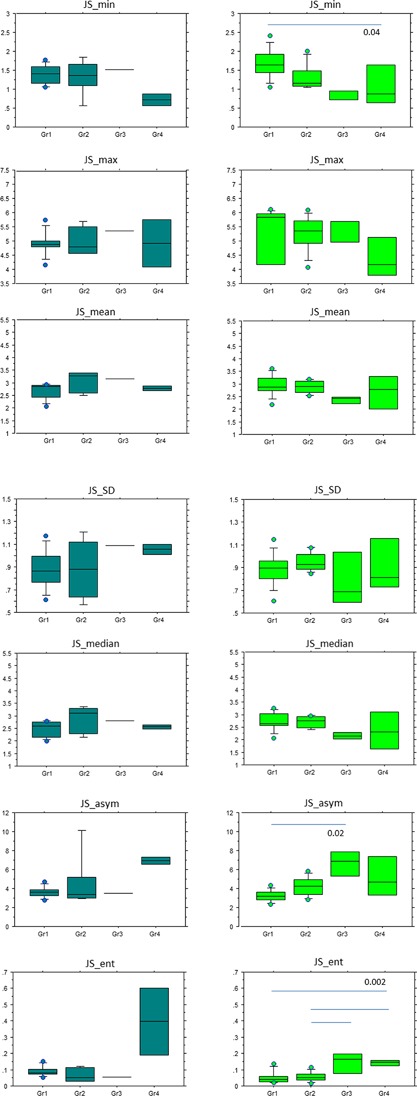
Box plots of JS_min, JS_max, JS_mean, JS_SD, JS_median, JS_asym, JS_ent values according to the meniscal grades of males (right): Gr1 (*n* = 8), Gr2 (*n* = 5), Gr3 (*n* = 1), Gr4 (*n* = 2) and females (left): Gr1 (*n* = 10), Gr2 (*n* = 8), Gr3 (*n* = 3), Gr4 (*n* = 4).

Figures 3, 4, and 5. report the distribution of statistical parameters derived from the 3D maps for males and females, separately, according to the KL (Fig. 3), Outerbridge (Fig. 4), and meniscal classifications (Fig. 5).

According to the Kellgren Lawrence classification, for males, (*n* = 27), JS_min, JS_max were significantly different between KL = 0 and KL = 3–4 with *p* = 0.02, and *p* = 0.001, respectively. For JS_max, the difference was also significant between KL = 0 with KL = 1 and KL = 2. In the population of females (*n* = 44), JS_min, JS_max, JS_mean, and JS_median, JS_ent were significantly different KL = 0 and KL = 3–4 with 0.04 < *p* < *0.004*. According to the Outerbridge classification, for males (*n* = 16), JS_min results were statistically significant between Gr1 and Gr4 with *p* = 0. 0008, JS_mean between Gr1 and Gr2, with *p* = 0.03, JS_asym between Gr1–Gr2 and Gr4 with *p* = 10^−4^, and JS_ent between Gr1–Gr2 and Gr4 with *p* = 0.04. For females (*n* = 25), JS_ent results were significantly different between Gr1 with Gr2, Gr3, and Gr4 with *p* = 0.0004. For the meniscal classification, in the population of females (*n* = 25), JS_min results were statistically different between Gr1 and Gr4 with *p* = 0.04, JS_asym between Gr1 and Gr3 with *p* = 0.02 and JS_ent between Gr1 with Gr4 and Gr2 with Gr3 and Gr4 with *p* = 0.002.

**Figure 6:**
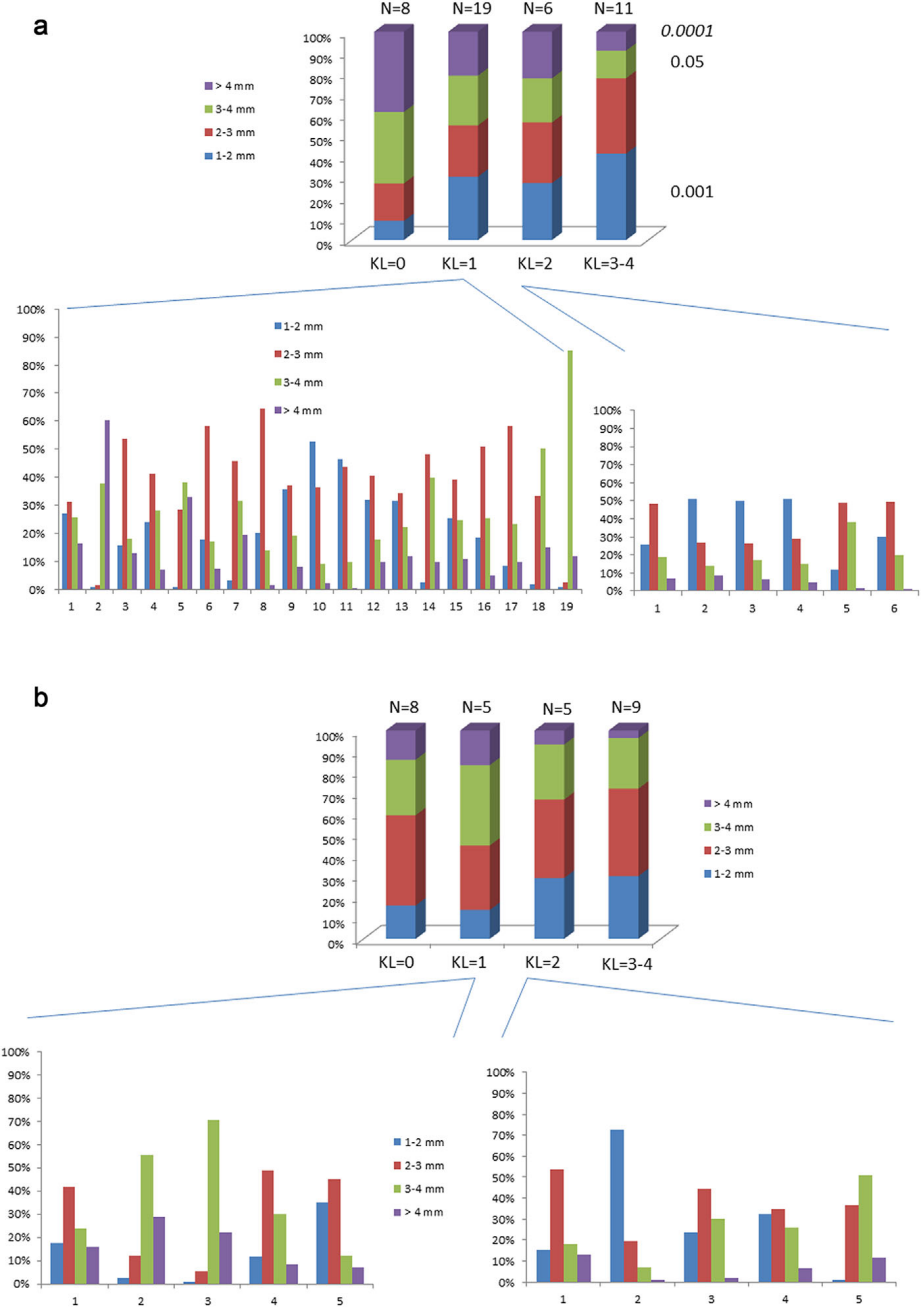
The mean JSW distributions (JSW_1–2 mm_, JSW_2–3 mm_, JSW_3–4 mm_, JSW_>4 mm_) from 3D maps according to the KL classification (a) for the female subgroup (*n* = 44) and (b) for the male subgroup (*n* = 27). For both groups, individual JSW distribution classified as KL = 1 and KL = 2.

In the subgroup of specimens from females, the details of JSW distribution (JSW_1–2 mm_, JSW_2–3 mm_, JSW_3–4 mm_, JSW_>4 mm_) found in different KL grades and the individual profiles for KL = 1 and KL = 2 are represented (Fig. 6). Values of JSW_1–2 mm_ were found in 9.8% in KL = 0 and in 41% in KL = 3‐4, values of JSW_> 4mm_ were found in 15.1% in KL = 0 and 3.3 % in KL = 3–4. The distributions of JSW_1–2 mm_ were found significantly different between KL = 0 and KL = 1 with KL = 3–4 (*p* = 0.001), the distributions of JSW_>4 mm_ were found significantly different between KL = 0, KL = 1 with KL = 3–4 and between KL = 0 with KL = 2 (*p* = 0.0001). The distributions of JSW_3–4 mm_ were found significantly different between KL = 0 with KL = 3–4 (*p* = 0.05).

In the population of females, there was a large heterogeneity of profiles in KL = 1, the majority of individuals (13/19) had a majority of JSW_2–3 mm_, (3/19) had a majority of JSW_3–4 mm_ and (2/19) a majority of JSW_1–2 mm,_ and finally (1/19) a majority of JSW_>4 mm_. For KL = 2, 50% of individuals has a majority of JSW_1–2 mm_ and the other 50% a majority of JSW_2–3 mm_. In the population of males, for KL = 1, the majority of individuals (3/5) had a majority of JSW_2–3 mm_, (2/5) had a majority of JSW_3–4 mm_. For KL = 2, (3/5) of individuals had a majority of JSW_2–3 mm,_ (1/5) of individuals had a majority of JSW_3–4 mm,_ and finally, (1/5) of individuals had a majority of JSW_1–2 mm._

The correlation coefficients between local measurements of cartilage thickness performed in three different sites at 10 µm of resolution are reported in Table 1. There were moderate correlation coefficients in most of JSW parameters with *r* values between −0.49 and +0.46 in the posterior area partially covered by the meniscus (0.002 < *p *< 0.001). The parameter JS_asym was constantly correlated with cartilage thickness measurements whatever the locations with *r* between −0.46 and −0.41 (0.01 < *p *< 0.002). The JS_median was significantly correlated with cartilage thickness in the central site site with *r* = 0.33, (*p* = 0.03), JS_ent was correlated with cartilage thickness in the peripheral site with *r* = −0.35 (*p* = 0.02).

**Table 1:** Pearson's Correlation Coefficients Among Statistical Parameters From the JSW Map and Cartilage Thickness Measured at Three Sampling Sites in the Medial Tibial Plateau: Central (Uncovered), Peripheral (Fully Covered), and Posterior (Partially Covered) Positions

*n* = 41	Posterior Position	Central Position	Peripheral Position
JS_vol	0.14	−0.25	0.06
JS_min	0.38^*^	0.22	0.27
JS_max	0.20	0.12	0.28
JS_mean	0.46^**^	0.27	0.25
JS_SD	0.09	−0.12	0.02
JS_median	0.45^**^	0.33^*^	0.27
JS_asym	−0.49^**^	−0.40^*^	−0.41^**^
JS_ent	−0.34^*^	−0.15	‐0.35^*^

JS, joint space; vol, volume; min, minimum; max, maximum; SD, standard deviation; asym, asymmetry; ent, entropy.

^*^ *p* = 0.05–0.01.

^**^ *p* = 0.01–0.001.

## DISCUSSION

The semi‐automated method presented in this article allows quantitative measurements of local variation of JS based on 3D high‐resolution computed tomography images. The majority of parameters were found interesting: JS_min, JS_max, JS_mean, JS_median, and JS_ent with differences between normal knee specimens defined by KL = 0 compared to OA knee specimens with KL = 3–4. The JS_min, JS_asym and JS_ent parameters were found to be different based on the Outerbridge and meniscal classifications between Gr1 with Gr3–4.

The 2D joint space narrowing assessment is still recommended for trials of structure modification.^4^ However, OA involves a complex arrangement of local thinning and thickening of cartilage thickness making necessary a complete assessment of knee compartments.^32^

The process of this technique presents some manual operations, first the determination of the threshold value used to binarize the images, then the choice of the antero‐posterior limits of the VOI, and finally the initialization of the snake contour. The highest variation coefficients were found for JS_max and JS_asym parameters, 0.17 mm and 0.21, respectively. The JS_asym variations are directly dependent on the calculation of JS_max values highly dependent on the map boundaries chosen by the operator. That could explain why JS_asym was found inconstantly different between groups defined by the different classifications. The high contrast of the bone component allows an easy segmentation of the joint space. The combination of the bone extraction by a thresholding method and the snake contour are complementary. If there are still remains of soft tissue in the case of an imperfect thresholding phase, it can be compensated for by the snake contour. Active contours by snake have already been used for cartilage segmentation on MRI images.^33^

On radiographs the most used parameter extracted was JS_min with an annual rate of progression of ∼0.2 mm for OA patients.^34^ In the present study, for this parameter, the difference between the two operators was about 10 times less than the supposed annual variation. The JS_asym was found constantly correlated with the cartilage thickness measured at three different sites and consequently presents a special interest. From a 3D quantitative analysis based on a semi‐automatic segmentation method at the wrist and metacarpal joints, JS_min and JS_asym have already been considered as the most interesting biomarkers in rheumatoid arthritis.^29^

The quantitative analysis based on CT images allowed us to have a real distribution of the JSW compared to an approach based on radiographs.^8^, ^9^, ^35^ The semi‐quantitative scoring of cartilage lesions based on CT arthrography has limitation with a low inter‐observer agreement.^20^ The presented method gives additional information compared to the KL classification on radiographs, which is based on a qualitative evaluation of the JSW variation and also on the presence of osteophytes. With a normal JS and a doubtful or definite osteophyte, the KL classification is KL = 1 and KL = 2, respectively. For knee specimens classified as KL = 1, 68% of them had a majority of JSW_2–3 mm_, and for them classified as KL = 2, 50% had JSW_2–3 mm_. These intermediate grades usually represent early knee OA but present a large variability of JSW distribution. Consequently, the 3D mapping of the JSW will be a useful tool able to characterize early OA. On radiographs, the measurement of JSW is highly dependent on the positioning of the patient in the X‐ray beam; however, with 3D imaging there are less positioning constraints. The main advantage of 2D radiographs is to be performed in a weight bearing position. The 2D quantification of JSW on CT slice was found to be more sensitive in a weight‐bearing position than in a non‐weight‐bearing position.^36^ We can imagine that the difference of the JS parameters extracted from the 3D map that we have obtained in our study would have been more pertinent between normal and OA groups if the images had been acquired in a weight‐bearing position.

The advantage of our method is to be validated against cartilage thickness performed on high‐resolution images (voxel size of 10 µm). The intra‐observer and inter‐observer precisions and the accuracy of the cartilage thickness measurements have already been performed and published in a study from our group. We have found that the imprecision of cartilage thickness measurements was 100 time less than the biological variations.^25^ Kijowski et al. have found quite low sensitivity for detecting the JS narrowing (about 46%) in the medial compartment from radiographs compared to arthroscopic finding from the articular surface.^7^ To test the reliability of JSW measurements in 2D radiographs as a predictor of cartilage thickness, Buckland Wright et al. compared their results with double contrast macro‐arthrograms where local cartilage measurements were performed. They have found Pearson coefficients greater than 0.91 in the medial compartment.^35^ The correlation coefficients found in the present study were moderated because there were no exact site‐matching between the two measurements; therefore our results are indicative and demonstrate the interest in and the reliability of these methods. The JS segmentation method tested here on the entire medial compartment corresponds to a situation as close as possible to the clinical situation.

Quantitative analysis of cartilage thickness was largely developed based on MRI images, but high field (3 or 7 Teslas) machines are necessary, and a resolution of at least 0.3 mm × 0.3 mm × 0.5 mm is necessary to obtain reliable cartilage‐thickness measurements.^13^ The method developed here on CT images has the advantage of being used on isotropic voxel images which is the best way to obtain exact Euclidean distances in all directions. The images provided by CT machines are widely used for dimensional metrology in many applications especially medical ones.^37^ The accuracy of cartilage defect measurements was found better on CT arthrography than MRI.^38^ For soft tissue visualization, meniscus, and cartilage visualization, CT will not replace MRI, nevertheless the very good contrast between bone structure and soft tissue that we can find using CT allows the use of pertinent segmentation methods. Contrary to MRI, the main drawback of our approach is the inability to differentiate cartilage and meniscus influence on JS narrowing which is known to be associated with both cartilage and meniscal damages.^39^ Indeed, the position and degeneration of meniscus have a great impact on joint space.^40^ We have found a parallel behavior of JS parameters between Outerbridge classification and meniscus grades except for JS_asym in the male population. In a previous study, with double contrast macro‐radiographs, it was demonstrated that in the medial compartment, articular cartilage damage of the tibia was markedly attached to meniscal damage contrary to femoral cartilage.^41^ One of the limitations of our study is that we performed only cartilage measurements at the tibia because it was demonstrated that tibial coverage by the meniscus and femur cartilage thickness explained 80% of the variability of joint space width.^42^ The Kellgren Lawrence classification is known to have low reproducibility and moderate sensitivity.^43^ For these reasons, semi‐automatic methods are usually performed in clinical studies, and one of the limitation of the present study is to be not compared with a semi‐automatic method performed on plain radiographs.

High‐resolution peripheral QCT systems are still not available for musculoskeletal‐extremity imaging but probably will be in the near future. The beam geometry of the system that we used did not allowed rapid scan, we need eight contiguous scans with a time duration about 20 min, the effective dose is less than 5 μSv per scan. With the same system, the effective dose at the wrist for a 16 min scan was 12.6 μSv.^29^

In a preliminary study, we have found that the segmentation method continues to have good performance on lower resolution images with a cone beam geometry acquisition.^28^ At the present time, new cone‐beam CT machines are already available on the market for musculoskeletal‐extremity imaging. The scans are performed in a weight‐bearing position with fast acquisition^36^ and provide good image quality for bone and an adequate quality for soft tissue.^44^ Their usefulness in knee OA diagnosis has already been demonstrated.^44^, ^45^

The cone‐beam CT technique has been identified to provide image data with isotropic spatial resolution and to support accurate JSW measurements manually performed on a coronal slice.^36^ For quantifying the tibiofemoral JS, different semi‐automatic methods have been tested on cone‐beam CT images.^46^, ^47^ High‐resolution cone‐beam CT systems with voxel size <100 µm for assessment of bone and joint health are under development.^48^

We have demonstrated that morphological analysis of the joint space performed on CT images with sufficiently high resolution is pertinent to describe indirectly tibial cartilage and meniscal impairments. The parameters JS_min, JS_asym, and JS_ent are the most interesting to characterize OA, and JS_asym despite a low reproducibility is a good predictor of the cartilage thickness. The JSW distributions based on a 3D map have a special significance in knee specimens classified KL = 1 and KL = 2 presenting a large heterogeneity of JSW. The JS morphology parameters measured in the medial compartment can be complemented by other geometrical measurements from CT images. This approach would potentially replace 2D radiographs and will benefit to new generation of CT systems to detect early stage of knee OA and it potentially be a tool to follow the progression of OA.

## AUTHORS’ CONTRIBUTIONS

CC participated in conception and design, provision of study materials, analysis and interpretation of the data and drafting of the manuscript. HM and RY participated in analysis and interpretation of the data. JDL conception and design, provision of study materials. CC takes responsibility for the manuscript content. All authors have read and approved the final manuscript.
